# Do individual psychological characteristics predict induction and generalization of nocebo and placebo effects on pain and itch?

**DOI:** 10.3389/fpsyt.2022.838578

**Published:** 2022-08-04

**Authors:** Lingling Weng, Antoinette I. M. van Laarhoven, Kaya J. Peerdeman, Andrea W. M. Evers

**Affiliations:** ^1^Health, Medical and Neuropsychology Unit, Faculty of Social and Behavioral Sciences, Leiden University, Leiden, Netherlands; ^2^Leiden Institute for Brain and Cognition, Leiden University, Leiden, Netherlands; ^3^Department of Psychiatry, Leiden University Medical Center, Leiden, Netherlands; ^4^Medical Delta, Leiden University, Technical University Delft, Rotterdam University, Rotterdam, Netherlands

**Keywords:** predictors, nocebo effects, placebo effects, pain, itch, pruritus, generalization

## Abstract

**Background:**

Nocebo and placebo effects, i.e., adverse or beneficial treatment effects, respectively, putatively due to expectancies can modulate pain and itch. These effects can generalize within the pain or itch modality. Predicting the induction and generalization of these effects can be helpful in clinical practice. This study aims to investigate whether psychological characteristics related to the fear-avoidance model predict the induction and generalization of nocebo and placebo effects on pain and itch in young healthy participants.

**Methods:**

Data from two previous experiments were analyzed. In Experiment 1, we induced nocebo and placebo effects on heat pain and tested generalization to pressure pain and to cowhage-evoked itch (*n* = 33 in a nocebo group, *n* = 32 in a placebo group). In Experiment 2, we induced nocebo effects on cowhage-evoked itch and tested generalization to mechanical itch and to mechanical touch (*n* = 44). Potential predictors were anxiety- and stress symptoms, attention to pain/itch, and pain/itch catastrophizing. Multiple regression analyses were performed.

**Results:**

For nocebo effects, none of the individual psychological characteristics significantly predicted induction of nocebo effects nor their generalization. For placebo effects, only less stress symptoms, lower attention to pain, and higher pain catastrophizing weakly predicted a stronger generalization of placebo effects from heat pain to pressure pain.

**Conclusion:**

The tested psychological characteristics may not play an important role in the induction and generalization of nocebo and placebo effects in healthy individuals. However, firm conclusions cannot be drawn with the current sample. Future studies should validate findings in larger and more diverse samples.

## Introduction

Placebo effects and nocebo effects, the beneficial and adverse treatment outcomes that cannot be ascribed to active treatments ingredients, respectively, can decrease and increase symptoms like pain and itch (1–3) by expectancy mechanisms. Expectancies can be effectively shaped by verbal suggestion (*via* providing explicit information) and classical conditioning (*via* repeatedly pairing a neutral stimulus with an unconditioned stimulus that naturally evokes a specific response) (2, 3). Recently, placebo and nocebo effects were found to generalize within the pain and itch modalities (4–6). This phenomenon is called response generalization, where similar placebo/nocebo effects can be found on perception of a novel stimulus that is different from the original stimulus for which placebo/nocebo effects were evoked (7). For instance, patients who experienced negative treatment outcomes may be prone to experience also similar negative treatment outcomes for similar symptoms, presumably mediated by expectancies. The susceptibility to placebo and nocebo effects as well as their generalization varies across individuals (8), making it difficult to harness them in clinical settings. It can be valuable to identify those individuals who are more sensitive to induction and generalization of placebo and nocebo effects.

Although mixed, evidence has shown that psychological characteristics related to the fear-avoidance model such as affective factors (including anxiety- and stress symptoms) and cognitive factors (including attention and catastrophizing) may be associated with placebo and nocebo effects on pain (1, 9–14), So far, most of what we know about the findings of predictors comes from the study of these effects on pain (11, 13–15). Only few studies explored the role of predictors in induction of placebo and nocebo effects on itch (12). Given the history of inconsistent findings on the predictors for placebo/nocebo effects and the paucity of studies on predicting these effects on itch, it is important to extend the current understanding of the relations between cognitive-affective factors and placebo/nocebo effects.

Cognitive-affective factors beyond expectancies may also influence generalization of placebo/nocebo effects from one symptom to similar symptoms. This is indirectly supported by research into fear generalization because of closely overlapping experimental procedures used when examining classical conditioning and generalization of (pain-related) fear and of placebo and nocebo effects (16, 17). Specifically, pain-related fear may arise as a by-product of the procedure of pain-related conditioning in placebo/nocebo effects, and one recent experimental study showed that pain-related fear can contribute to nocebo hyperalgesia (18). Therefore, it is reasonable to assume that the factors that influence fear generalization such as affect (e.g., anxiety- and stress symptoms) (19, 20) and cognitions (e.g., attention) (21), may also be associated with generalization of placebo/nocebo effects. However, no studies have explored predictors for generalization of placebo and nocebo effects on somatosensory sensations yet. Understanding whether and how psychological characteristics are involved in the induction and generalization of placebo/nocebo effects could be clinically relevant to foster the efficacy of positive treatment outcomes and minimize the severity of negative treatment outcomes within or across symptoms.

Our aims were to explore whether psychological characteristics can predict the induction and generalization of placebo and nocebo effects on somatosensory sensations in young healthy participants. Specifically, we explore if anxiety- and stress symptoms, as well as attention, and catastrophizing can predict (1) induction and generalization of nocebo effects (primary objective), (2) induction and generalization of placebo effects (secondary objective), (3) expected nocebo and placebo effects as well as generalization (exploratory objective). Given indirect support from the fear-avoidance model (22, 23), we would expect that these cognitive-affective factors may positively predict nocebo effects (and generalization) and negatively predict placebo effects (and generalization). To this end, in two different experiments [from which the findings on nocebo and placebo effects have been published in separate articles (4, 24)] we first measured individual psychological characteristics with self-report questionnaires. In the first experiment, we consecutively induced nocebo and placebo effects on heat pain and tested generalization of nocebo and placebo effects to pressure pain and to cowhage-evoked itch (4). In the second experiment, we induced nocebo effects on cowhage-evoked itch and tested generalization of nocebo effects to mechanical itch and to mechanical touch (24).

## Materials and methods

A brief summary of the two experiments (i.e., the information of participants and the experimental designs) can be found below. The procedures have been extensively described in our previous publications (4, 24), and are briefly repeated in Supplementary Appendix Method.

### Participants

The sample size calculations were conducted for the main (placebo/nocebo) outcomes of two experiments (4, 24). Specifically, each group (placebo or nocebo) in experiment 1 would require 34 participants (4), and experiment 2 would require 44 participants (24). *Post-hoc* power analyses suggest that these sample sizes are sufficient to detect large effect sizes (*f*^2^ > 0.35) for multiple regression analyses with 4 predictors (α = 0.05, power = 0.8). However, sample sizes of >25 should be sufficient to conduct multiple regressions (25). All participants (English-speaking) were between 18 and 35 years old. All participants were recruited *via* an online recruitment system (Sona Systems, Tallinn, Estonia) and through flyers posted in and around the university. Exclusion criteria were: current physical or mental illness, suffering from chronic itch (≥6 weeks), currently using medication or psychoactive drugs, being pregnant or lactating. Additionally, experiment 1 also excluded participants who were suffering from chronic pain (≥6 months), and experiment 2 excluded participants when they experienced spontaneous itch ≥3 on a 0 (not itch at all)-10 (worst itch imaginable) numerical rating scale (NRS) at the start of the testing session or cowhage insensitivity. Both experiments were approved by the Psychology Research Ethics Committee of Leiden University (CEP19-1205/571 and CEP18-1218/491). The experiments were conducted at Leiden University, the Netherlands. All participants provided their written informed consent. A data-blind preregistration for the current study was published at AsPredicted (#71238.^1^ None of the currently reported analyses had been conducted prior to pre-registration).

### Study designs

Both experiments used a within-subject design. Noteworthy, participants received neither verbal suggestions nor conditioning regarding the stimuli used for investigating *generalization*. All stimuli were applied in a pseudorandom order.

#### Experiment 1

**FIGURE 1:**
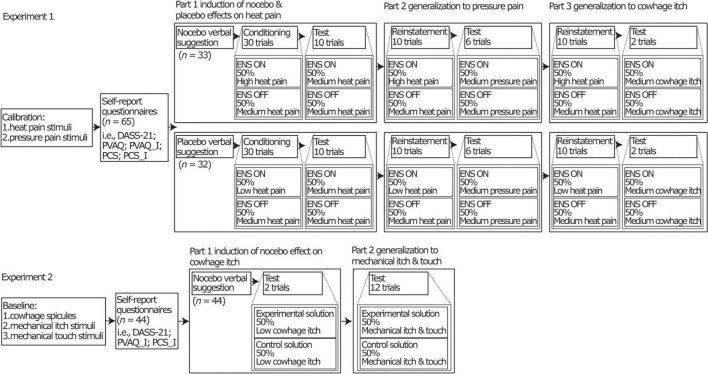
Overview of the full design of the two separate experiments. For experiment 1, “ENS” was functioned as a placebo treatment. “ON” and “OFF” indicated the sham (de)activation of the ENS device. “ON” represents an experimental trial and “OFF” represents a control trial. The ENS device was a transcutaneous electrical nerve stimulation (TENS) device (model EM80, Beurer, Germany). Participants rated their pain intensities on a 0 (no pain at all)-10 (worst pain imaginable) numerical rating scale (NRS). Low (NRS 0.5-2), moderate (NRS 3-4.5), and high (NRS 5.5-7) heat pain intensities were individually calibrated. Moderate pressure pain intensity (NRS 3-4.5) was individually determined. For experiment 2, “experimental solution” was served as a nocebo treatment. “Experiment solution” represents an experiment trial and “control solution” represents a control trial. Throughout both experiments, participants received all stimuli in half of experimental trials and in half of control trials in all phases. DASS-21, The 21-item version of the Depression Anxiety Stress Scale; PVAQ, The Pain Vigilance and Awareness Questionnaire; PCS, The Pain Catastrophizing Scale; PVAQ-I, itch-adjusted version of the PVAQ; PCS-I, itch-adjusted version of the PCS. For more details of the design for two experiments see (4, 24).

The experiment had two independent groups (i.e., nocebo group and placebo group). During the experiment, we first induced nocebo and placebo effects on heat pain, and then tested generalization to pressure pain and to cowhage-evoked itch. All participants underwent a design consisting of 3 parts (see Figure 1). Part 1 comprised an induction phase and a test phase, where participants either received a negative expectation induction (nocebo group) or a positive expectation induction (placebo group) by verbal suggestion and conditioning (see Supplementary Appendix Method) regarding heat pain stimuli and tested on heat pain stimuli (see Supplementary Appendix Method). Part 2 comprised a short version of the conditioning in part 1 (*Reinstatement* in Figure 1) and a test phase to test generalization to pressure pain stimuli (see Supplementary Appendix Method). Part 3 comprised the same short version of the conditioning in part 1 (*Reinstatement* in Figure 1) and a test phase to test generalization to cowhage-evoked itch (see Supplementary Appendix Method).

#### Experiment 2

We first induced nocebo effects on cowhage-evoked itch and then tested generalization to mechanical itch and to mechanical touch. The design included 2 parts. Part 1 comprised an induction and a test phase, where participants received a negative expectation induction by verbal suggestion (see Supplementary Appendix Method) on cowhage-evoked itch and tested on cowhage-evoked itch. Part 2 comprised a test phase to test generalization to mechanical itch and mechanical touch (see Supplementary Appendix Method).

### Assessment of predictors

Psychological characteristics, specifically anxiety-, stress-, depressive symptoms, attention to pain/itch, pain/itch catastrophizing were measured with the questionnaires described below. In experiment 1, all mentioned questionnaires were administered. In experiment 2, all questionnaires except those pertaining specifically to pain were administered. All questionnaires were administered in English and completed using Qualtrics (Qualtrics, Provo, United States) on a desktop computer in the lab before administering somatosensory stimuli in both experiments.

####  Anxiety-, stress-, and depressive symptoms

The 21-item version of the Depression Anxiety Stress Scale (DASS-21) was used to measure the frequency and severity of experiencing negative emotions over the previous week. The scale consists of subscales of anxiety (e.g., “I was aware of dryness of my mouth”), depression (e.g., “I felt that life was meaningless”), and stress (e.g., “I found it hard to wind down”). Each item was rated on a Likert scale from 0 (did not apply to me at all) to 3 (applied to me very much, or most of the time). Seven items per scale were summed and doubled to be equivalent to the full DASS version. The scores of each subscale theoretically range from 0 to 42, with higher scores indicating greater state anxiety, stress, and depression, respectively (26, 27). Cronbach’s alpha of the subscales in both experiments ranged from 0.69 to 0.78, except from the subscale depression in experiment 1 in the placebo group (Cronbach’s alpha = 0.52).

#### Attention to pain

The Pain Vigilance and Awareness Questionnaire (PVAQ) was used to measure the frequency of self-reported attentional habits with a focus on pain and changes in pain. This scale consists of 16 items, e.g., “I am very sensitive to pain.” Each item was rated on a Likert scale from 0 (never) to 5 (always). All items were summed, with a theoretical range from 0 to 80, with higher scores indicating a higher focus on pain sensations (28). Cronbach’s alpha of the PVAQ was 0.84 in experiment 1 and 0.85 in experiment 2.

#### Attention to itch

The PVAQ was adjusted to pertain itch (PVAQ-I) by only replacing the word “pain” with “itch” for all items, e.g., “I am very sensitive to itch” (29). Cronbach’s alpha of the PVAQ-I was 0.83 in experiment 1 and 0.86 in experiment 2.

#### Pain catastrophizing

The Pain Catastrophizing Scale (PCS) was used to measure catastrophizing about pain experienced in daily life. This scale consists of 13 items, e.g., “I become afraid that the pain will get worse.” Each item was rated on a Likert scale from 0 (not at all) to 4 (all the time). All items were summed, with a theoretical range from 0 to 52, with higher scores indicating more pain catastrophizing (30). Cronbach’s alpha of the PCS was 0.85 in experiment 1 and 0.93 in experiment 2.

#### Itch catastrophizing

The PCS was adjusted to pertain itch (PCS-I) by only replacing the word “pain” with “itch” for all items, e.g., “I become afraid that the itch will get worse” (29, 31). Cronbach’s alpha of the PCS-I was 0.84 in experiment 1 and 0.92 in experiment 2.

### Statistical analysis

All analyses were performed using R (Version 3.6.3, Vienna, Austria) for Windows. Nocebo and placebo effects were defined as the difference in scores between experimental and control trials during the test phases in both experiments (4, 24). Furthermore, we defined generalization responders as participants who reported higher sensation scores in experimental trials in the testing generalization phases in the nocebo group or lower scores in the placebo group when compared to control trials. Due to a low-reliability of the DASS-21’s subscale depression, this subscale was removed as predictor from all analyses. Assumption checks included normality, linearity, homoscedasticity, and multicollinearity. All assumptions were met in this study. Influential values were checked by Cook’s distance (>0.5 considered as influential values, see Supplementary Appendix Figures). In case of influential values, the main outcomes would be conducted with and without influential values. Given the small sample size, regression analyses were conducted with bootstrapping (2,000 samples with reporting 95% confidence intervals (CIs)]. The statistically significant level was set at *p* < 0.05.

To check whether psychological characteristics were related to the induction and generalization of nocebo and placebo effects and to check the intercorrelations between predictors for each model, Pearson correlation coefficients (normal distribution) were calculated.

To examine the primary objective of exploring predictors for the induction and generalization of nocebo effects, multiple regression analyses were performed in which the psychological characteristics (i.e., anxiety-, stress symptoms, attention to pain/itch, pain/itch catastrophizing) were entered into the model simultaneously (i.e., forced entry) as predictors. Dependent outcomes were nocebo effects on heat pain, nocebo effects on cowhage-evoked itch, generalization of nocebo effects to pressure pain, to cowhage-evoked itch, and to mechanical itch and touch. Note that, in experiment 2, we observed that mechanical stimuli induced impure sensations at baseline (i.e., the mechanical touch filaments evoked itch and the mechanical itch filaments did not evoke itch at baseline). Therefore, we selected those filaments that evoked either touch or itch at baseline for each individual (“individualized mechanical touch/itch filaments”) to assess the nocebo effects evoked in the test phase and included these outcomes as dependent variables in present analyses (24). Further, note that psychological characteristics related to pain were not used to predict dependent outcomes related to itch, and vice versa for itch. An overview of the specific predictors and dependent outcomes is reported in Supplementary Appendix Table 1.

To examine the secondary objective of exploring predictors for placebo effects, the same method and predictors as described in the primary objective were used, except that the dependent outcomes were placebo effects on heat pain as well as generalization of placebo effects to pressure pain and cowhage-evoked itch (see Supplementary Appendix Table 1).

To examine the exploratory objectives of exploring predictors for *expected* itch and pain (referred to *expected nocebo and placebo effects* in the remainder), the same method and predictors as described in the primary objective were used, except that the dependent outcomes were the *expected* itch and pain intensities.

## Results

### Sample characteristics

In experiment 1, 33 participants were included in the nocebo group and 32 participants in the placebo group. In experiment 2, 44 participants were included. Due to the sensitivity check in which those participants were excluded who did not perceive the baseline stimuli as intended, e.g., mechanical itch stimuli not evoking itch (24), 29 participants were included in the analyses of the models related to mechanical touch, and 39 participants in the analyses of the models related to mechanical itch. Participants’ demographics and spontaneous fatigue/pain/itch levels are reported in Supplementary Appendix Table 2.

### Induced and generalized nocebo and placebo effects

Induction and generalization of nocebo and placebo effects were previously reported (4, 24). A summary of descriptive results of all stimuli scores by group and trial type are reported in Supplementary Appendix Tables 3, 4. In short, in experiment 1, both nocebo and placebo effects were significantly induced on heat pain as hypothesized. As also hypothesized, nocebo and placebo effects significantly generalized from heat pain to pressure pain, but contrary to our hypothesis they did not generalize to cowhage-evoked itch. In experiment 2, nocebo effects were significantly induced on cowhage-evoked itch as hypothesized. As also hypothesized, nocebo effects from cowhage-evoked itch significantly generalized to mechanical itch, but contrary to our hypothesis nocebo effects did not generalize to mechanical touch. In both experiments, at least 60% of participants were classified as generalization responders for each generalization effect, despite a lack of generalization effects across modalities at the group level. Frequencies of participants showing generalization per effect are reported in Supplementary Appendix Table 5.

### Predictors and intercorrelations

Tables 1, 2 display an overview of mean, standard deviations, observed range, and intercorrelations of dependent outcomes and the relevant predictors in both experiments. Regarding nocebo effects, the correlation coefficients showed that none of the predictors was significantly associated with induction and generalization of nocebo effects. Regarding placebo effects, only stress symptoms were significantly associated with generalization of placebo effects to pressure pain (*r* = −0.39, *p* = 0.03).

**TABLE 1:** Mean ± SD and intercorrelations of predictors and dependent outcomes in the nocebo and the placebo group in experiment 1.

Experiment 1	M ± SD	Observed range (min-max)	1	2	3	4	5	6	7	8
**Nocebo group (*n* = 33)**										
1. Induction of heat pain	0.4 ± 0.6	−1.2–1.5								
2. Generalization to pressure pain	0.5 ± 1.3	−3.6–3.3	0.08							
3. Generalization to cowhage-evoked itch	0.6 ± 2.3	−6–7	−0.10	0.24						
4. Anxiety	4.3 ± 5.3	0–24	0.03	0.12	0.12					
5. Stress	8.6 ± 6.7	0–30	0.15	0.08	−0.28	0.27				
6. Pain catastrophizing	13.3 ± 7.0	0–29	−0.01	0.34	n/a	0.26	0.18			
7. Attention to pain	34.0 ± 10.1	19–56	−0.21	0.25	n/a	0.00	0.07	0.46**		
8. Itch catastrophizing	10.1 ± 6.1	0–31	n/a	n/a	0.08	0.09	0.16	n/a	n/a	
9. Attention to itch	24.9 ± 10.7	0–51	n/a	n/a	0.27	0.31	0.16	n/a	n/a	0.37*

**Placebo group (*n* = 32)**										

1. Induction of heat pain	0.6 ± 0.7	−2–0.9								
2. Generalization to pressure pain	0.8 ± 1.0	−3.3–1.1	−0.04							
3. Generalization to cowhage-evoked itch	0.1 ± 2.3	−4–6.3	0.00	0.24						
4. Anxiety	2.9 ± 4.1	0–16	−0.07	−0.23	−0.01					
5. Stress	6.9 ± 5.1	0–20	−0.25	−0.3*	0.00	0.58***				
6. Pain catastrophizing	13.3 ± 9.4	1–43	−0.11	0.09	n/a	0.26	0.28			
7. Attention to pain	35.5 ± 10.4	12–63	−0.12	−0.32	n/a	0.28	0.09	0.53**		
8. Itch catastrophizing	8.2 ± 7.3	0–26	n/a	n/a	−0.06	0.17	−0.03	n/a	n/a	
9. Attention to itch	26.1 ± 10.1	6–51	n/a	n/a	−0.16	0.29	−0.02	n/a	n/a	0.32

*p < 0.05, **p < 0.01, ***p < 0.001 (two-tailed); SD, standard deviation. n/a, not applicable.

Individual psychological characteristics related to pain were not used to predict dependent outcomes related to itch, and vice versa for itch. The dependent outcomes, i.e., induction of heat pain, generalization to pressure pain, and generalization to cowhage-evoked itch, were calculated as the scores of experimental trials minus control trials for each stimulus in the nocebo group and control trials minus experimental trials in the placebo group. The scores of anxiety, stress, and depression subscales (DASS-21) theoretically range from 0 to 42; the scores of attention to pain and attention to itch (PVAQ and PVAQ-I) theoretically range from 0 to 80; the scores of pain catastrophizing and itch catastrophizing (PCS and PCS-I) theoretically range from 0 to 52. Note that these results of subscale depression was removed due to the low reliability of the depression subscale.

**TABLE 2:** Mean (M) ± SD and intercorrelations of predictors and dependent outcomes in experiment 2 (*n* = 44).

Experiment 2 (nocebo group)	M ± SD	Observed range (min-max)	1	2	3	4	5	6
1. Induction of cowhage-evoked itch	0.8 ± 2.4	−5.7–7						
2. Generalization to mechanical itch	0.3 ± 0.9	−1.1–4.1	0.06					
3. Generalization to mechanical touch	0.4 ± 1.1	−2.6–2.6	0.30	0.51**				
4. Anxiety	4.3 ± 4.9	0–26	−0.05	−0.04	0.10			
5. Stress	7.5 ± 6.0	0–26	0.02	−0.09	−0.09	0.60***		
6. Itch catastrophizing	9.7 ± 7.3	0–28	0.13	−0.18	−0.07	0.07	0.21	
7. Attention to itch	30.0 ± 10.7	8–46	0.01	0.03	−0.04	−0.02	−0.06	0.28

** p < 0.01, *** p < 0.001 (two-tailed); SD, standard deviation. n/a, not applicable.

The dependent outcomes, i.e., induction of cowhage-evoked itch, generalization to mechanical itch, and generalization to mechanical touch, were calculated as the scores of experimental trials minus control trials for each stimulus in the test phases. The scores of anxiety, stress, and depression subscales (DASS-21) theoretically range from 0 to 42; the scores of attention to itch (PVAQ-I) theoretically range from 0 to 80; the scores of itch catastrophizing (PCS-I) theoretically range from 0 to 52. Note that these results of subscale depression was removed due to the low reliability of the depression subscale.

### Regression analyses

Table 3 displays the results of regression analyses regarding induction and generalization of nocebo and placebo effects. The results of regression analyses regarding *expected* nocebo and placebo effects are listed in Supplementary Appendix Table 6.

**TABLE 3:** An overview of multiple regression analyses *via* forced entry to predict induction of nocebo and placebo effects on heat pain and their generalization to pressure pain and to cowhage-evoked itch in experiment 1 (*n* = 33 in the nocebo group, *n* = 32 in the placebo group), and to predict induction of nocebo effects on cowhage-evoked itch and their generalization to mechanical itch and to mechanical touch in experiment 2 (*n* = 44).

Nocebo effects									Placebo effects										
		Induction of heat pain			Generalization to pressure pain			Generalization to cowhage itch			Induction of heat pain			Generalization to pressure pain			Generalization to cowhage itch		
		β	*SE*_*a*_	95% CI	β	*SE*_*a*_	95% CI	β	*SE*_*a*_	95% CI	β	*SE*_*a*_	95% CI	β	*SE*_*a*_	95% CI	β	*SE*_*a*_	95% CI
**Experiment 1**																			
	Anxiety	0	0.02	−0.04,0.04	0.01	0.05	−0.06,0.13	0.06	0.11	−0.32,0.02	0.03	0.05	−0.08,0.12	0.03	0.05	−0.09,0.11	0.04	0.12	−0.14,0.28
	Stress	0.01	0.02	−0.02,0.07	0	0.04	−0.07,0.08	−0.13	0.09	−0.12,0.13	−0.05	0.03	−0.11,0.02	−0.1	0.03*	−0.18, −0.05	−0.02	0.09	−0.26,0.12
	Pain catastrophizing	0.01	0.02	−0.02,0.05	0.05	0.04	−0.03,0.12	n/a	n/a		0	0.02	−0.03,0.04	0.05	0.02*	0.01,0.09	n/a	n/a	
	Attention to pain	−0.01	0.01	−0.04,0.01	0.02	0.03	−0.02,0.08	n/a	n/a		−0.01	0.01	−0.04,0.02	−0.05	0.02*	−0.09, −0.01	n/a	n/a	
	Itch catastrophizing	n/a	n/a		n/a	n/a		0.01	0.06	−0.05,0.14	n/a	n/a		n/a	n/a		−0.01	0.11	−0.20,0.22
	Attention to itch	n/a	n/a		n/a	n/a		0.06	0.04	−0.01,0.12	n/a	n/a		n/a	n/a		−0.04	0.05	−0.14,0.07

Full model		Adj. *R*^2^ = −0.05			Adj. *R*^2^ = 0.01			Adj. *R*^2^ = 0.01			Adj. *R*^2^ = −0.04			Adj. *R*^2^ = 0.32			Adj. *R*^2^ = −0.12		
		F(4, 28) = 0.61			F(4, 28) = 1.06			F(4, 28) = 1.69			F(4, 27) = 0.67			F(4, 27) = 4.67			F(4, 27) = 0.20		
		*p* = 0.661			*p* = 0.394			*p* = 0.180			*p* = 0.616			*p* = 0.005			*p* = 0.94		

**Nocebo effects**																			
	
		**Induction of cowhage itch**			**Generalization to mechanical itch**			**Generalization to mechanical touch**											
		β	*SE*_*a*_	95% CI	β	*SE*_*a*_	95% CI	β	*SE*_*a*_	95% CI									

**Experiment 2**																			
	Anxiety	−0.04	0.1	−0.29,0.14	0	0.03	−0.06,0.11	0.04	0.06	−0.09,0.15									
	Stress	0.02	0.07	−0.11,0.18	−0.01	0.03	−0.06,0.05	−0.03	0.03	−0.08,0.02									
	Itch catastrophizing	0.04	0.05	−0.04,0.16	−0.02	0.03	−0.08,0.03	0	0.03	−0.06,0.04									
	Attention to itch	−0.01	0.03	−0.08,0.07	0.01	0.02	−0.02,0.04	0	0.02	−0.05,0.04									

Full model		Adj. *R*^2^ = −0.08			Adj. *R*^2^ = −0.07			Adj. *R*^2^ = −0.12											
		F(4, 39) = 0.22			F(4, 34) = 0.54			F(4, 24) = 0.28											
		*p* = 0.928			*p* = 0.844			*p* = 0.886											

* p < 0.05, *β* is the standardized regression coefficient. n/a, not applicable, SE_a._, bootstrap standard error of the mean. CI, bootstrapped confidence interval. The results of all models in both experiments used the raw values. Depressive symptom was not included in the models due to low reliability of the depression subscale. Due to the sensitivity check, 29 participants were included in the analyses of the models related to mechanical touch, and 39 participants in the analyses of the models related to mechanical itch.

Regarding the primary objective concerning nocebo effects, in line with the results from the correlations, multiple regression analyses indicated that the studied psychological characteristics predicted neither induction of nocebo effects on heat pain and cowhage-evoked itch, nor generalization of nocebo effects within modalities (i.e., from heat pain to pressure pain and from cowhage-evoked itch to mechanical itch) or across modalities (i.e., from heat pain to cowhage-evoked itch and from cowhage-evoked itch to mechanical touch) (Table 3). Influential values were observed in the model of generalization of nocebo effects to cowhage-evoked itch, but removal of the influential values did not lead to different results.

Regarding the secondary objective concerning placebo effects, multiple regression analyses showed that lower stress symptoms (*β* = −0.1, 95% CI [−0.18, −0.05]), less attention to pain (*β* = −0.05, 95% CI [−0.09, −0.01]), and higher pain catastrophizing (*β* = 0.05, 95% CI [0.01, 0.09]), predicted stronger generalization of placebo effects to pressure pain (full model: F_(4,27)_ = 4.67, *p* = 0.005, Adj. *R*^2^ = 0.32) (Table 3).

Regarding the exploratory objective concerning expected nocebo and placebo effects, multiple regression analyses showed that lower itch catastrophizing (*β* = −0.15, 95% CI [−0.28, −0.04]) and higher attention to itch (*β* = 0.07, 95% CI [0.01, 0.14]) predicted higher expectancies of nocebo effects on cowhage-evoked itch (generalization) (full model: F_(4,28)_ = 3.27, *p* = 0.025, Adj. *R*^2^ = 0.22) (Supplementary Appendix Table 6). Similar analyses showed that less attention to itch (*β* = −0.06, 95% CI [−0.12, −0.02]) alone predicted higher expectancies of nocebo effects on mechanical sensations (generalization) (full model: F_(4_,_39)_ = 1.72, *p* = 0.166, Adj. *R*^2^ = 0.06) (Supplementary Appendix Table 6).

## Discussion

The current study aimed to explore predictors for induction and generalization of nocebo and placebo effects within and across pain and itch modalities. Our results showed that anxiety-, stress symptoms, pain/itch catastrophizing, and attention to pain/itch did not significantly predict, with relatively small confidence intervals, induction of nocebo and placebo effects. Regarding generalization, only lower stress symptoms, lower attention to pain, and higher pain catastrophizing weakly predicted a stronger generalization of placebo effects from heat pain to pressure pain. These findings and their implications should be interpreted with caution, considering the sample was limited in size and consisted of young healthy individuals.

Regarding nocebo effects, the findings that the psychological characteristics did not predict nocebo effect induction are in line with several previous studies indicating the lack of significant associations between psychological characteristics and nocebo effects (1, 9, 14, 32). Moreover, no significant predictors were found for generalization of nocebo effects within and across the pain and itch modalities. This may be partly caused by our target sample of young healthy individuals who have, unsurprisingly, low levels of negative affect and cognitions. It should be noted that nocebo effects were not found to generalize across modalities. Therefore, replication is necessary before drawing a conclusion. The exploratory analyses of the prediction of participants’ expectancies showed that lower itch catastrophizing and higher attention to itch predicted higher expectancies of nocebo effects on cowhage-evoked itch (generalization). As the overall pooled associations were small and the (directions of) predictors were not consistently found for generalization across the two experiments, these findings should be interpreted with caution. From a hypothesis-generating perspective, the current study paves the way to further explore potential predictors of generalization of nocebo effects.

Regarding placebo effects, the findings that the psychological characteristics did not predict placebo effect induction contrasts with some previous research with comparable sample sizes e.g., (11, 33). However, two recent studies with large cohorts yielded mixed results, with one study (*N* = 397) reporting negative associations between negative affect (including anxiety-, and stress symptoms) and placebo effects (10) and one reporting (*N* = 624) null associations (14). Further research herein may examine possible interactions between multiple predictors and explore other potential predictors (e.g., fear). Regarding generalization of placebo effects, there are some indications for psychological characteristics that may explain small parts of the variance. Specifically, stronger generalization of placebo effects within the pain modality may be predicted by lower stress symptoms, less attention to pain, and higher pain catastrophizing. One potential explanation could be that people with lower stress symptoms and less attention to symptoms, may tend to focus on positive information and avoid harmful information (34, 35). However, the result also showed that higher pain catastrophizing may be relevant to a stronger generalization of placebo effects, which contrasts with theory (36) and previous research (37). As these predictors only explained a small part of the variance, no firm conclusions can be drawn from these findings. Further research is warranted to validate these findings. Moreover, the exploratory results did not suggest that psychological characteristics predict expectancies of the induction and generalization of placebo effects. One possible explanation is that the psychological characteristics measured in this study may be less relevant in the facilitation/inhibition of positive expectancies (38, 39). More research is warranted, as a better understanding of individual responses could foster the efficacy of positive treatment outcomes.

### Limitations and suggestions for future studies

First, given the limited sample size and the inclusion of only young healthy participants, variances in the characteristics could have been restricted, and false negative findings might have occurred. Besides, representativeness of the demographics and psychological characteristics could limit the generalizability of the current findings to the general population or patient populations. Further studies should include more variance in characteristics such as age and health status (40, 41). Besides, although our sample size met a minimum requirement (*N* = 25) for multiple regressions with multiple predictors (25) and our study was of exploratory, hypothesis-generating nature, only large effects may be detected with this small sample and thus results should be interpreted with caution. Future research with larger cohorts is required, for instance in the forms of meta-analyses on individual data. Second, considering the low reliability of the depression subscale (depressive symptoms were removed from all analyses), further studies may use other questionnaires such as Positive and Negative Affect Schedule e.g., (42). Also, the generally low levels of cognitive-affective factors could not provide a comprehensive insight into their predictive value. It may be helpful to include participants at different baseline levels of cognitive-affective factors. Next to self-report measurements, experimental research directly manipulating factors such as anxiety- and stress symptoms and assessing effects on induction and generalization of nocebo and placebo effects seems to be currently lacking. Third, the lack of generalization of nocebo effects across modalities at the group level may have affected the current results. However, psychological characteristics may still help to distinguish individuals who tend to generalize and those who do not at the individual level, although this study did not provide a clear pattern. Finally, it is common that prediction research, including ours, only included few potential predictors at once. However, it appears suboptimal to account for only few factors to predict nocebo and placebo effects as well as their generalization, especially in clinical settings. Future studies are recommended to not only examine multiple psychological characteristics at once, but also to combine these characteristics with other factors such as personality traits, e.g., (15, 43) genetic variants, e.g., (44, 45) doctor-patient relationships, e.g., (46, 47) treatment history, e.g., (48) and various contextual variables e.g., (49), to get a comprehensive multifaceted structure of predicting nocebo and placebo effects.

### Suggestions for future research

Some suggestions need to be discussed. On top, assessing changes in dynamic individual characteristics, such as state anxiety and state fear, before versus after the nocebo and placebo manipulations could provide more insight into the underlying dynamics of nocebo and placebo effects as well as their generalization. Second, as different mechanisms are supposed to underlie nocebo and placebo effects (3), it is recommended to assess different predictors for nocebo and placebo effects, e.g., anxiety for nocebo effects and optimism for placebo effects (32, 33). Another recommendation to advance the field is to systematically test theoretical models such as the fear-avoidance model e.g., (22) and a predictive coding framework regarding symptom perception e.g., (50). Finally, including patient samples would be an important next step. For instance, patients with chronic itch due to atopic dermatitis appear to be more sensitive to nocebo-like effects on itch than healthy individuals (51, 52). Assessing the predictors in patients’ treatment outcomes as well as subsequent treatment outcomes would contribute to identifying patients who are sensitive to nocebo and placebo effects. This could eventually provide individualized interventions to increase treatment effectiveness.

## Conclusion

This study suggests that the psychological characteristics may not (or only weakly) predict the induction and generalization of nocebo and placebo effects in young healthy individuals. Given the current restrictions to the sample, however, it cannot be ruled out that these characteristics do play a significant role in placebo and nocebo effects on pain and itch and their generalization. The current study can be a starting point for further exploring the relevance of these predictors for generalization of nocebo and placebo effects. Exploring the predictors for nocebo and placebo effects as well as their generalization would contribute to helping treatment outcomes in the clinic and establishing individualized treatments schemes, thereby helping increase the success of treatments.

## Data availability statement

Data not included in the article/Supplementary material will be made available through Dataverse (https://dataverse.nl/dataverse/leidenuniversity). Further inquiries also can be directed to the corresponding author.

## Ethics statement

The studies involving human participants were reviewed and approved by the Psychology Research Ethics Committee of Leiden University. The participants provided their written informed consent to participate in this study.

## Author contributions

LW analyzed the data and drafted the manuscript. All authors have critically revised, edited, and approved the final manuscript.

## Footnotes

1
https://aspredicted.org/blind.php?x=/BN5_TRN
