# Supplementary material for: Do individual psychological characteristics predict induction and generalization of nocebo and placebo effects on pain and itch?
Source: Front Psychiatry. 2022 Aug 4;13:838578. doi: 10.3389/fpsyt.2022.838578 (PMC9386339; doi:10.3389/fpsyt.2022.838578)
Supplement: Supplementary file 1 [file Data_Sheet_1.docx]

Supplementary Material

Note: This is the supplementary material to the article “Do individual psychological characteristics predict induction and generalization of nocebo and placebo effects on pain and itch?” by Weng L, Laarhoven AIM, Peerdeman KJ, Evers AWM. It contains a more detailed description of the methods of each experiment (Appendix Method), figures of cook’s distance per effect (Appendix Figures), and tables with extra information (Appendix Tables).

**Appendix Method** (a more detailed description of the methods of each experiment)

**Placebo and nocebo manipulations**

In experiment 1, participants in the placebo group received verbal suggestions that activating an ENS device (ON, Figure 1) can decrease heat pain whereas participants in the nocebo group were informed that activating the ENS device can increase heat pain. The text ‘ON’ on a computer screen indicated the sham activation of the ENS device – for the control trials, the text ‘OFF’ appeared on the screen. The ENS device was an actual transcutaneous electrical nerve stimulation (TENS) device (model EM80, Beurer, Germany). The device served as a placebo and did not effectively work in this experiment. Conditioning and reinstatement phases were achieved by surreptitiously applying during half of the trials at low/high heat pain intensity with the sham activation of the ENS device (experimental trial) and half at medium heat pain intensity without the sham activation of the ENS device (control trial), in a pseudorandom order. In the test phases, all stimuli were delivered at medium intensity, half of which with the sham activation of the ENS device and half without the sham activation of the ENS device.

In experiment 2, all participants received verbal suggestions that a compound called “Cyclosol” (experimental solution; with a label on the botte showing “Cyclosine Solution”) can increase cowhage-evoked itch and the solution without “Cyclosol” (control solution; with a label on the bottle showing “Hydro Solution”) does not affect itch. One drop of either “Cyclosol” or control solution was topically applied to and rubbed onto the participants’ forearms with a Q-tip in the areas where the stimuli would subsequently be applied. The solution with ”Cyclosol” served as a nocebo treatment in this experiment, and both solutions were actually simply water. Of all stimuli, half were tested with the experimental solution and the other half with the control solution (see Fig.1).

**Pain induction and assessment**

*Heat pain induction.* A TSA-II neurosensory analyzer for sensory testing (TSA-II; Medoc Advanced Medical Systems, Ramat Yishai, Israel) was used to induce heat pain to the middle of the volar forearms with a 3 x 3 cm thermode probe. Low (NRS 0.5-2), moderate (NRS 3-4.5), and high (NRS 5.5-7) heat pain intensities were individually calibrated.

*Pressure pain induction.* A handheld algometer (Pain Diagnostic & Treatment, Italy) with a 1 cm diameter probe was used to induce pressure pain to the thenar eminence of the hand (potential range: 0-10 kg). Moderate pressure pain intensity (NRS 3-4.5) was individually determined.

*Psychophysics:* Throughout experiment 1, pain intensities were rated on an NRS ranging from 0 (no pain at all) to 10 (worst pain imaginable). Experienced pain intensities were rated after each pain stimulus. Expected pain intensities were rated before every 5 heat pain trials and before every 3 pressure pain trials in the test phases. In experiment 2, no pain stimuli were given.

**Cowhage application and assessment**

In experiment 1, around 45 cowhage spicules were used to induce a moderate itch intensity. Participants verbally reported their experienced itch intensity every 10 seconds for 3 minutes on the itch NRS and their expected itch intensity once before each application of cowhage spicules by using the same itch NRS. In experiment 2, around 25 spicules were applied to the volar surface of the forearm to induce a low to moderate itch intensity. Experienced itch scores were rated continuously for 4 minutes (sampled every 5 seconds) on a digital itch Visual Analogue Scale (VAS, ranging from 0 (no itch at all) to 10 (worst itch imaginable)) on an eVAS app (Aalborg University, Denmark) installed on a tablet (Lenovo TB2-X30F, Beijing, China), and expected itch intensities were rated once before each application of cowhage spicules by using the same digital VAS.

**Mechanical stimulation and assessment**

In experiment 2, Semmes-Weinstein von Frey filaments (North Coast Medical, Gilroy, USA) were used to induce mechanical touch and itch sensations to the volar surface of the forearm near the wrist. The filaments with the following forces were selected based on previous research and/or piloting20,21: 4.08, 4.17, 4.31, 5.07, 5.18, and 5.46 mN. Throughout experiment 2, experienced itch scores were rated on a digital itch NRS by using Qualtrics (Qualtrics, Provo, Utah, United States) on a desktop. Expected itch intensities were rated on the same NRS after applying each solution, yet before applying mechanical stimuli.

**Appendix Figures**

Note: the figures show the histogram of standardized residuals and cook’s distance (>0.5 as influential values) for each model.

1.
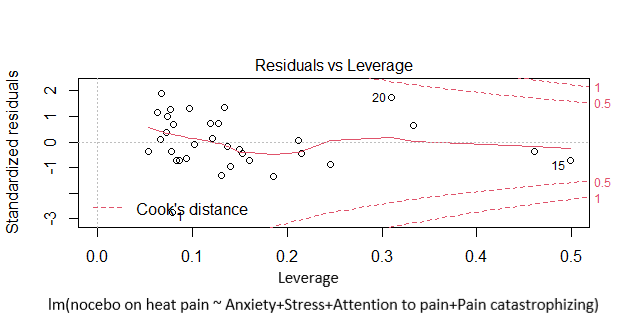
Nocebo effects on heat pain


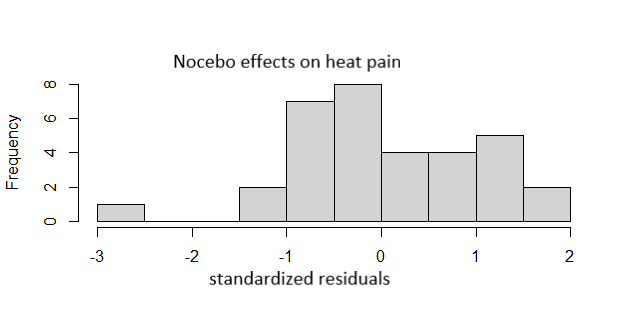


1.
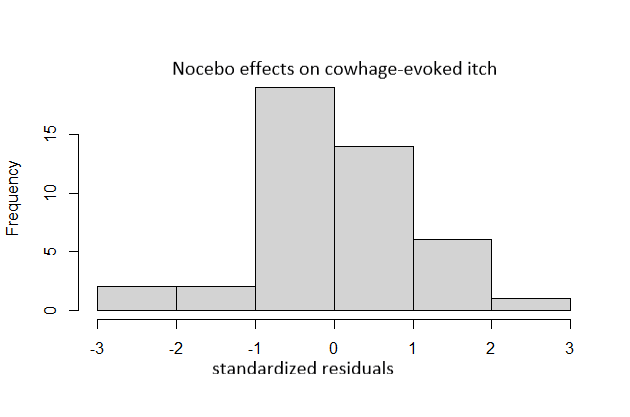

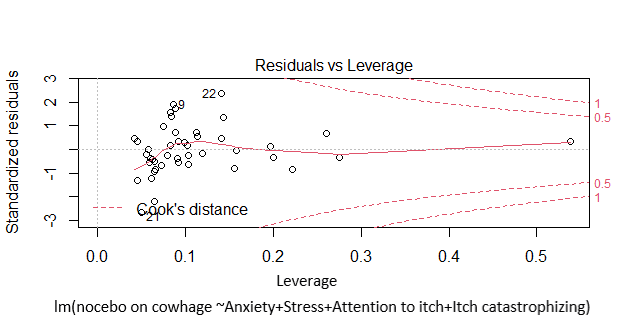
Nocebo effects on cowhage-evoked itch
2. Generalization of nocebo effects from heat pain to pressure pain


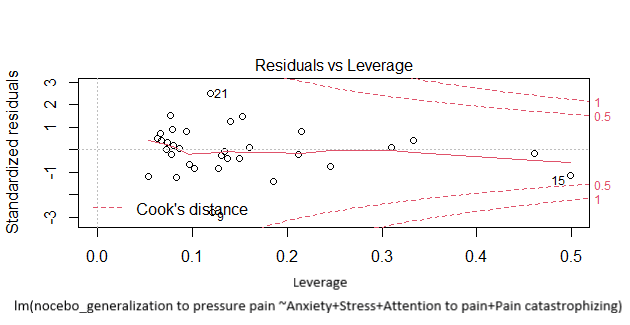

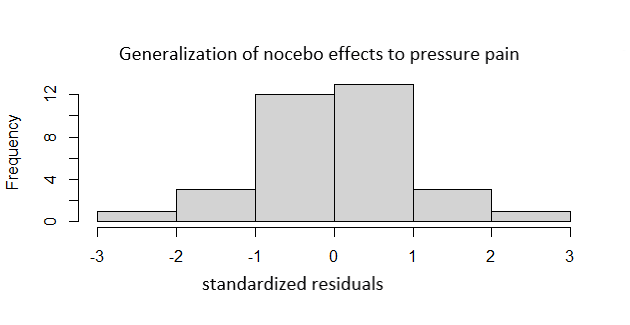


1.
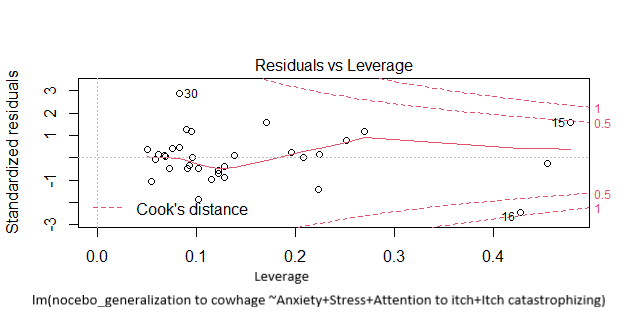
Generalization of nocebo effects from heat pain to cowhage-evoked itch


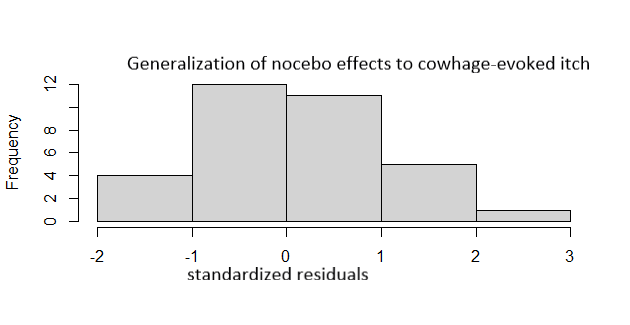


1.
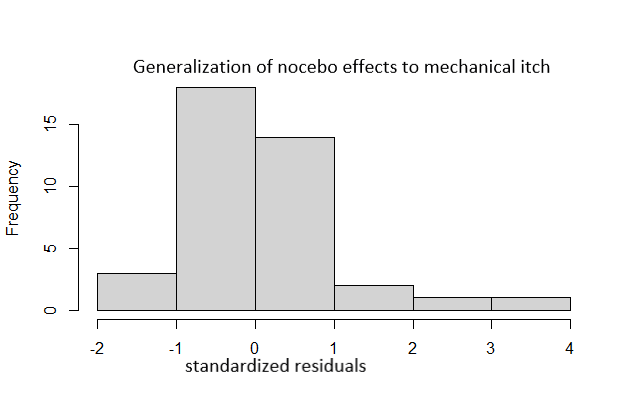

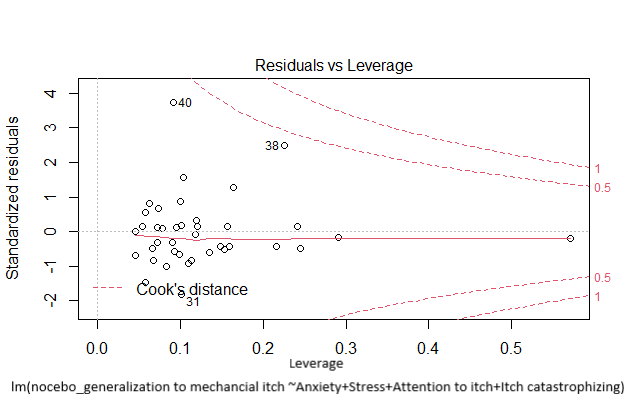
Generalization of nocebo effects from cowhage-evoked itch to mechanical itch
2.
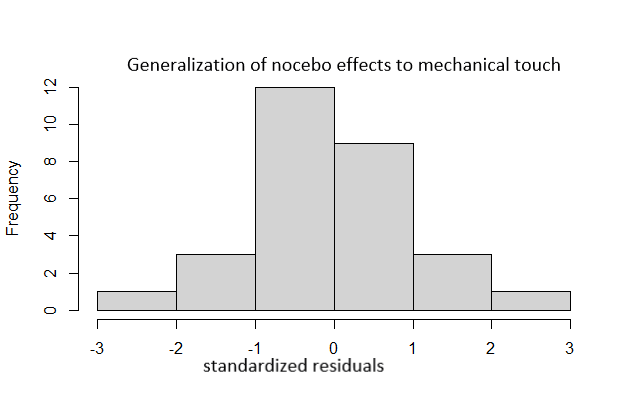

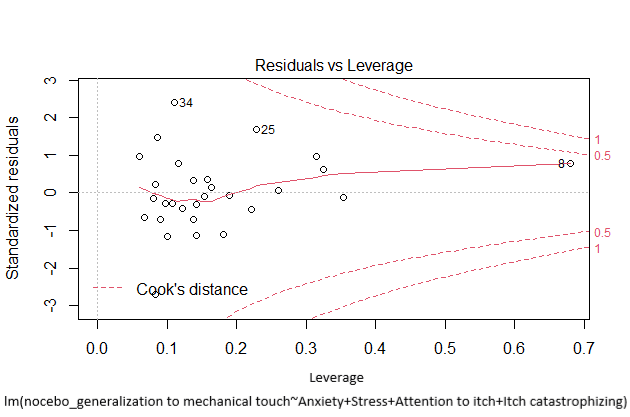
Generalization of nocebo effects from cowhage-evoked itch mechanical touch
3. Placebo effects on heat pain


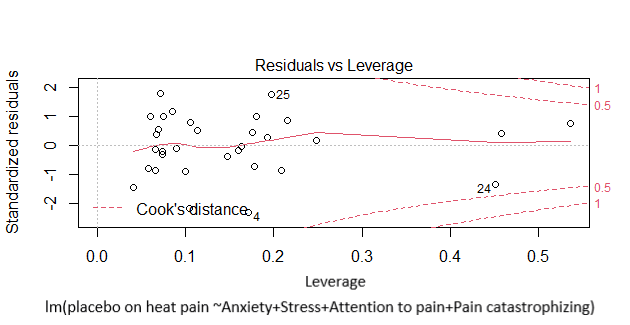

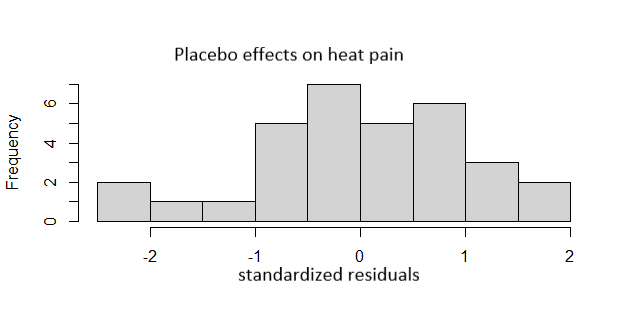


1.
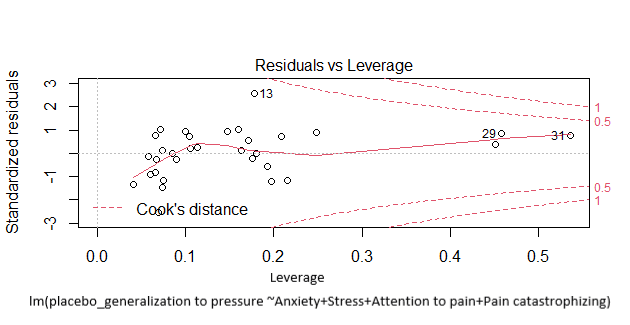
Generalization of placebo effects from heat pain to pressure pain


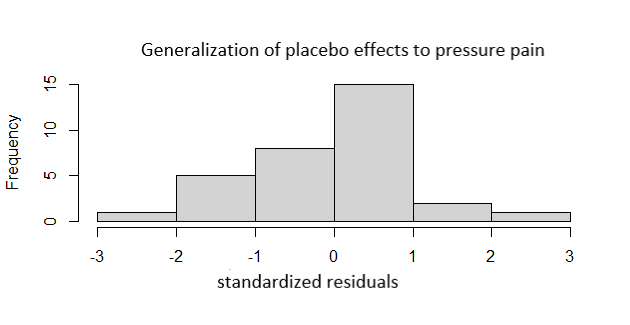


1. Generalization of placebo effects from heat pain to cowhage-evoked itch


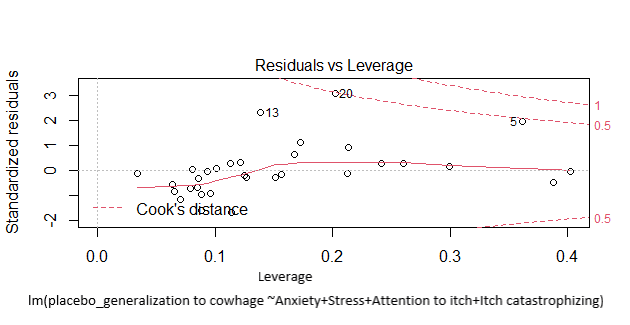

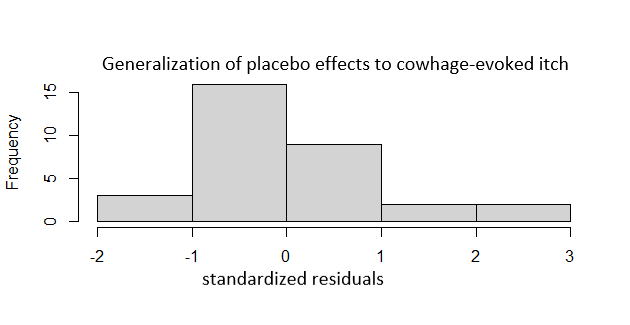


1. Expected nocebo effects on heat pain


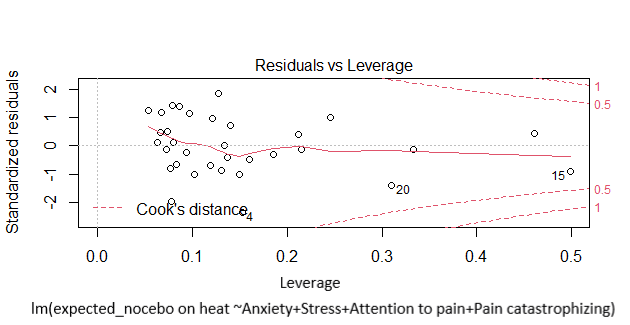

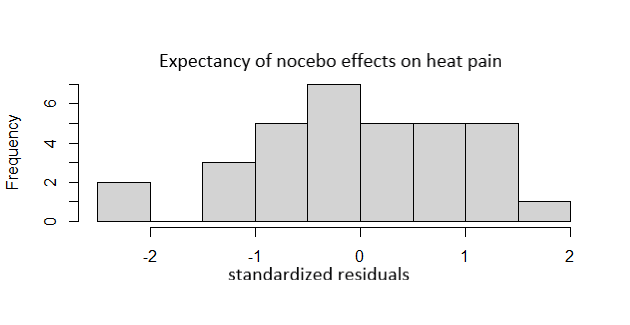


1. Expected nocebo effects on cowhage-evoked itch


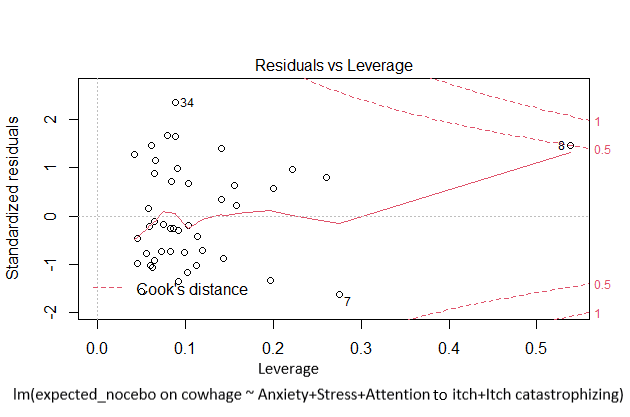


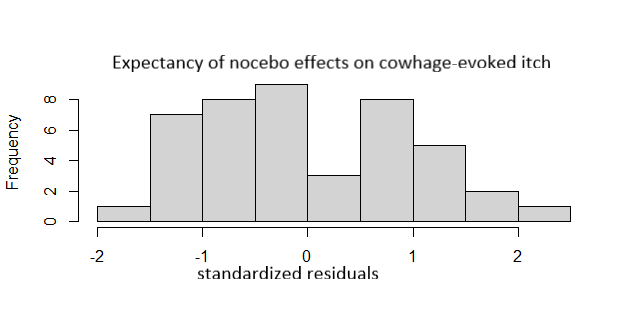


1. Expected generalization of nocebo effects from heat pain to pressure pain


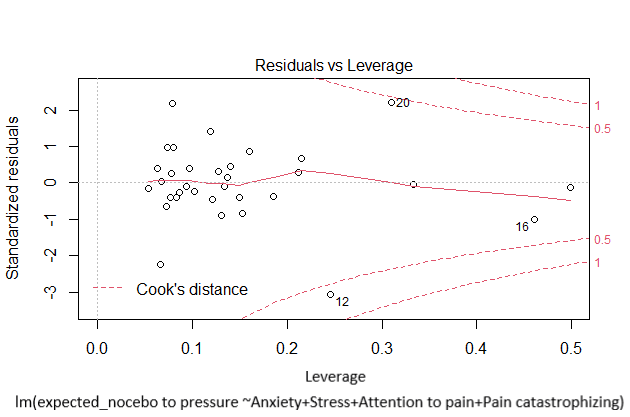

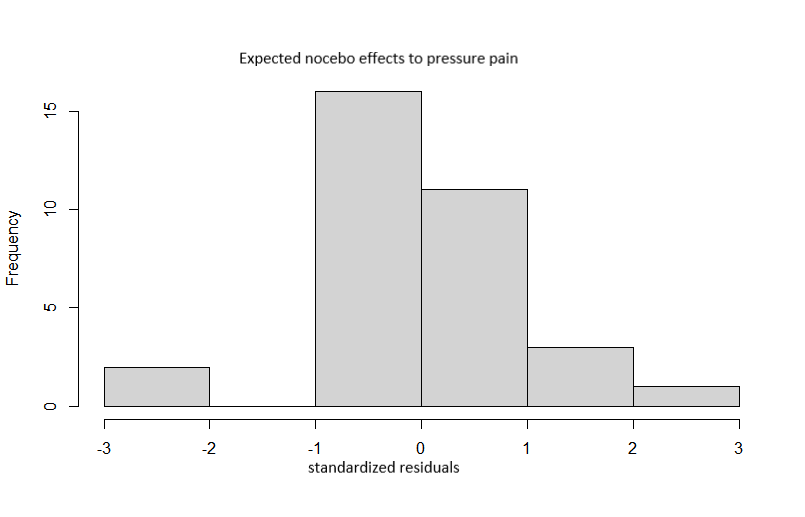


1.
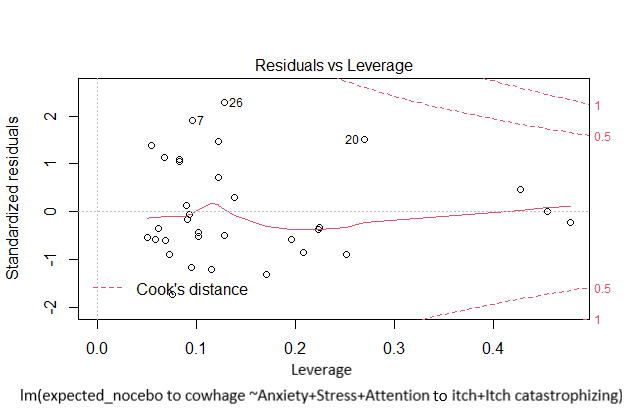

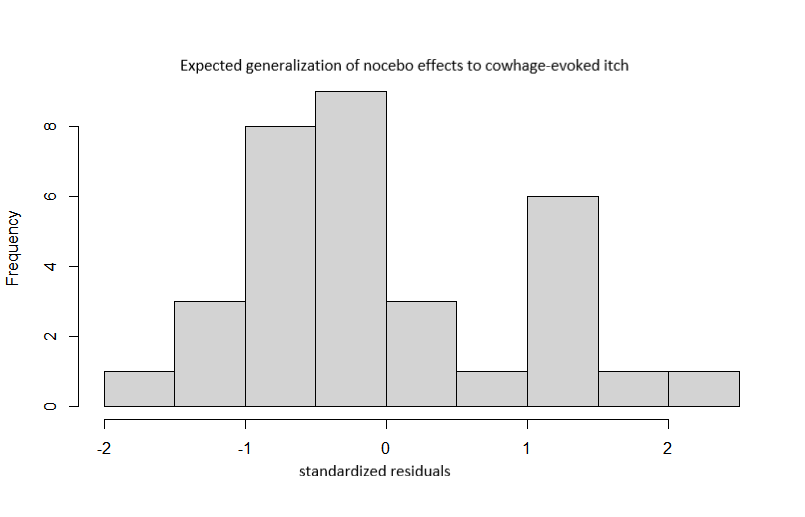
Expected generalization of nocebo effects from heat pain to cowhage-evoked itch
2. Expected generalization of nocebo effects from cowhage-evoked itch to mechanical filaments


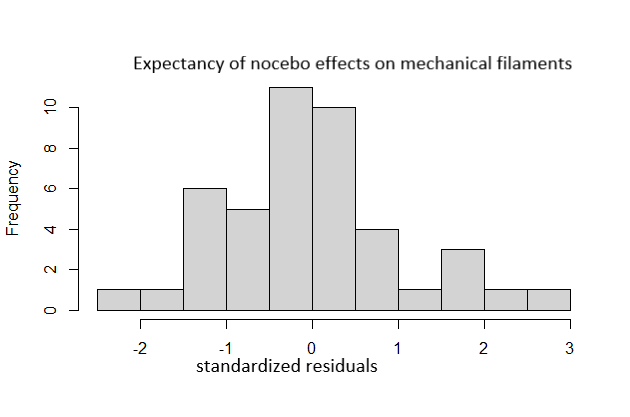

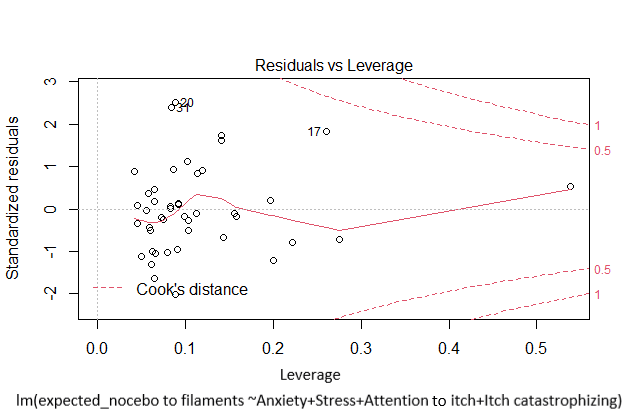


**Appendix Tables**

**Table 1.** Overview of the predictors and dependent outcomes in experiment 1 (*n* = 33 in the nocebo group, *n* = 32 in the placebo group) and experiment 2 (*n* = 44). The induction and generalization of nocebo outcomes are the primary outcome, and the induction and generalization of placebo outcomes are the secondary outcome of this research.

|  | **Experiment 1** | | | **Experiment 2** | | |
| --- | --- | --- | --- | --- | --- | --- |
| **Outcomes**  **Predictors** | **Induction of nocebo and placebo effects on** | **Generalization of nocebo and placebo effects on heat pain to** | **Generalization of nocebo and placebo effects on heat pain to** | **Induction of nocebo effects on** | **Generalization of nocebo effects on cowhage-evoked itch to** | **Generalization of nocebo effects on cowhage-evoked itch to** |
| Anxiety  Depression  Stress  (DASS-21) | heat pain | pressure pain | cowhage-evoked itch | cowhage-evoked itch | mechanically evoked itch | mechanically evoked touch |
| Attention to pain (PVAQ) | heat pain | pressure pain | n/a | n/a | n/a | n/a |
| Pain catastrophizing (PCS) | heat pain | pressure pain | n/a | n/a | n/a | n/a |
| Attention to itch (PVAQ_I) | n/a | n/a | cowhage-evoked itch | cowhage-evoked itch | mechanically evoked itch | mechanically evoked touch |
| Itch catastrophizing (PCS_I) | n/a | n/a | cowhage-evoked itch | cowhage-evoked itch | mechanically evoked itch | mechanically evoked touch |

Note: DASS-21, the 21-item version of the Depression Anxiety Stress Scale; PVAQ, the Pain Vigilance and Awareness Questionnaire; PCS, the Pain Catastrophizing Scale; PVAQ_I, itch-adjusted version of the PVAQ; PCS_I, itch-adjusted version of the PCS. n/a, not applicable.

**Table 2.** Demographics and spontaneous fatigue/pain/itch levels in both experiments.

|  | Experiment 1 | | Experiment 2 (N = 44) |
| --- | --- | --- | --- |
|  | Nocebo group (N = 33) | Placebo group (N = 32) |  |
| Age in years, M (SD) | 21.6 (3.0) | 22.0 (3.3) | 22.3 (3.6) |
| Women (%) | 25(76) | 24(75) | 33(75) |
| Education, n(%) |  |  |  |
| secondary education | 14(42.4) | 10(31.3) | 6 (13.6) |
| tertiary education | 19(57.6) | 20(62.5) | 37 (84.1) |
| other | 0(0) | 2(6.3) | 1 (2.3) |
| Baseline level, M (SD) |  |  |  |
| fatigue | 3.2(1.9) | 3.0(1.8) | 1.9 (1.6) |
| itch | 0.5(0.9) | 0.1(0.4) | 0.2 (0.4) |
| pain | 0.8(1.0) | 0.7(1.0) | 0.2 (0.6) |

*n*, sample size; M, mean; SD, standard deviation. Participants rated their spontaneous levels of fatigue, pain, and itch from 0 (no pain/fatigue/itch at all) to 10 (worst pain/fatigue/itch imaginable) at baseline.

| Experiment 1 | Experienced rating | |  |  |  |  | Expected rating | |  |  |  |  |
| --- | --- | --- | --- | --- | --- | --- | --- | --- | --- | --- | --- | --- |
|  | Nocebo group | |  | Placebo group | |  | Nocebo group | |  | Placebo group | |  |
|  | Conditioned | Control | Nocebo effects | Conditioned | Control | Placebo effects | Conditioned | Control | Nocebo effects | Conditioned | Control | Placebo effects |
| Heat pain | 3.3 ± 1.9 | 2.9 ± 1.8 | **0.4 ± 0.6***** | 2.6 ± 1.4 | 3.2 ± 1.4 | **0.6 ± 0.7***** | 5.5 ± 1.6 | 3.5 ± 2.0 | **2.0 ± 1.9***** | 1.3 ± 1.2 | 3.7 ± 1.6 | **2.4 ± 1.8***** |
| Pressure pain | 4.3 ± 1.5 | 3.8 ±1.5 | **0.5 ± 1.3**** | 2.6 ± 1.3 | 3.4 ± 1.2 | **0.8 ± 1.0***** | 4.1 ± 1.6 | 3.3 ± 1.6 | **0.7 ± 2.5*** | 2.0 ± 1.2 | 3.0 ± 1.2 | **1.0 ± 1.3***** |
| Cowhage-evoked itch | 3.9 ± 2.3 | 3.3 ± 2.3 | 0.6 ± 2.3 | 3.2 ± 2.3 | 3.3 ± 2.6 | 0.1 ± 2.3 | 4.3 ± 1.7 | 3.1 ± 1.7 | **1.3 ± 2.0**** | 1.8 ± 1.4 | 3.6 ± 1.7 | **1.8 ± 2.1***** |

**Table 3.** Mean ± SD for the experienced and expected levels of pain and itch scores on average evoked during the conditioned and the control trials used for assessing nocebo and placebo effects on heat pain and their generalization to pressure pain and to cowhage-evoke itch in experiment 1 (*n* = 33 in nocebo group, *n* = 32 in placebo group). For more details see.^4^

* *p* < 0.05, ***p* < .01, *** *p* < 0.001. SD, standard deviation. Note: nocebo effects were calculated as the scores of the conditioned trials minus the control trials for each stimulus and placebo effects as the control trials minus the conditioned trials for each stimulus. Only 1 expected itch rating was obtained for all mechanical stimuli (i.e., mechanical itch and mechanical touch combined) in experiment 2. Participants rated pain and itch scores from 0 (no pain/itch at all) to 10 (worst pain/itch imaginable). Mechanical itch and mechanical touch were induced by the individualized filaments that were individually selected to induce either itch or no itch at baseline.

**Table 4.** Mean ± SD for the experienced and expected levels of itch scores evoked during the nocebo and the control trials used for assessing nocebo effects on cowhage-evoked itch and their generalization to mechanical itch and to mechanical touch in experiment 2 (*n* = 44). For more detail see.^24^

| Experiment 2 | Experienced rating | |  | Expected rating | |  |
| --- | --- | --- | --- | --- | --- | --- |
|  | Nocebo | Control | Nocebo effects | Nocebo | Control | Nocebo effects |
| Cowhage-evoked itch | 2.2 ± 2.1 | 1.4 ± 1.5 | **0.8 ± 2.4*** | 4.1 ± 2.4 | 1.4 ± 1.6 | **2.7 ± 2.1***** |
| Mechanical itch | 1.4 ± 1.4 | 1.1 ± 1.0 | **0.4 ± 0.9*** | 3.5 ± 2.0 | 1.6 ± 1.4 | **1.9 ± 1.8***** |
| Mechanical touch | 1.9 ± 1.8 | 1.5 ± 1.5 | 0.3 ± 1.0 |  |  |  |

* *p* < 0.05, ***p* < .01, *** *p* < 0.001. SD, standard deviation. Note: nocebo effects were calculated as the scores of the conditioned trials minus the control trials for each stimulus. Only 1 expected itch rating was obtained for all mechanical stimuli (i.e., mechanical itch and mechanical touch combined) in experiment 2. Participants rated itch scores from 0 (no itch at all) to 10 (worst itch imaginable). Mechanical itch and mechanical touch were induced by the individualized filaments that were individually selected to induce either itch or no itch at baseline.

**Table 5.** Number of participants who responded to generalization of nocebo and placebo effects (called responders in the table) on experiment 1 (n = 33 in the nocebo group, n = 32 in the placebo group) and who responded to generalization of nocebo effects on experiment 2 (n = 44). Due to sensitivity check in experiment 2, 29 participants were included in the analyses of the model related to mechanical touch, and 39 participants in the analyses of the model related to mechanical itch.

|  | Responders on experiment 1, *n*(%) | | Responders on experiment 2, *n*(%) |
| --- | --- | --- | --- |
|  | Nocebo effects | Placebo effects |  |
| Generalization to pressure pain | 22(66.7) | 26(81.3) | n/a |
| Generalization to cowhage-evoked itch | 20(60.6) | 20(62.5) | n/a |
| Generalization to mechanical itch | n/a | n/a | 27(69.2) |
| Generalization to mechanical touch | n/a | n/a | 19(65.5) |

**Table 6.** An overview of multiple regression analysis via forced entry to predict expectancies of induction of nocebo and placebo effects on heat pain and expectancies of these effects on pressure pain (generalization) and on cowhage-evoked itch (generalization) in experiment 1 (*n* = 33 in the nocebo group, *n* = 32 in the placebo group), and to predict expectancies of induction of nocebo effects on cowhage-evoked itch and expectancies of nocebo effects on mechanical stimuli (generalization) in experiment 2 (*n* = 44).

|  |  | **Expected nocebo effects** | | | | | | | | | | |  | |  | |  | **Expected placebo effects** | | | | | | | | | | | | | | | | | | | | | | |
| --- | --- | --- | --- | --- | --- | --- | --- | --- | --- | --- | --- | --- | --- | --- | --- | --- | --- | --- | --- | --- | --- | --- | --- | --- | --- | --- | --- | --- | --- | --- | --- | --- | --- | --- | --- | --- | --- | --- | --- | --- |
|  |  | **Induction of heat pain** | | |  | **Generalization to pressure pain** | | |  | **Generalization to cowhage itch** | | | | | | |  | **Induction of heat pain** | | | | | | |  | | **Generalization to pressure pain** | | | | | |  | | **Generalization to cowhage itch** | | | | | |
|  |  | *β* | *SE_a_* | 95%CI |  | *β* | *SE_a_* | 95%CI |  | *β* | | | *SE_a_* | | 95%CI | |  | *β* | | | *SE_a_* | | 95%CI | |  | | *β* | | *SE_a_* | | 95%CI | |  | | *β* | | *SE_a_* | | 95%CI | |
| **Experiment 1** |  |  |  |  |  |  |  |  |  |  | | |  | |  | |  |  | | |  | |  | |  | |  | |  | |  | |  | |  | |  | |  | |
|  | **Anxiety** | .04 | .07 | -.09, .20 |  | -.01 | .08 | -.15, .23 |  | .01 | | | .07 | | -.11, .16 | |  | -.09 | | | .13 | | -.33, .18 | |  | | -.10 | | .09 | | -.25, .05 | |  | | -.04 | | .13 | | -.03, .18 | |
|  | **Stress** | -.01 | .05 | -.15, .09 |  | .01 | .09 | -.13, .29 |  | .08 | | | .06 | | -.06, .19 | |  | .06 | | | .08 | | -.14, .19 | |  | | .00 | | .07 | | -.14, .12 | |  | | -.02 | | .08 | | -.18, .15 | |
|  | **Pain catastrophizing** | -.05 | .06 | -.16, .07 |  | .05 | .09 | -.18, .21 |  | n/a | | | n/a | |  | |  | .03 | | | .05 | | -.09, .13 | |  | | .01 | | .03 | | -.04, .08 | |  | | n/a | | n/a | |  | |
|  | **Attention to pain** | .02 | .04 | .02, -.00 |  | .01 | .07 | -.13, .18 |  | n/a | | | n/a | |  | |  | -.03 | | | .05 | | -.12, .06 | |  | | .00 | | .03 | | -.05, .05 | |  | | n/a | | n/a | |  | |
|  | **Itch catastrophizing** | n/a | n/a |  |  | n/a | n/a |  |  | **-.16** | | | .06* | | -.27, -.04 | |  | n/a | | | n/a | |  | |  | | n/a | | n/a | |  | |  | | .06 | | .05 | | -.06, .15 | |
|  | **Attention to itch** | n/a | n/a |  |  | n/a | n/a |  |  | **.07** | | | .03* | | .02, .15 | |  | n/a | | | n/a | |  | |  | | n/a | | n/a | |  | |  | | .00 | | .04 | | -.08, .09 | |
| Full model | | Adj. *R^2^* = -.11  F(4, 28) = 0.23  *p* = .92 | | |  | Adj. *R^2^* = -.12  F(4, 27) = 0.18  *p* = .948 | | |  | Adj. *R^2^* = .22  F(4, 28) = 3.27  *p* = .025 | | | | | | |  | Adj. *R^2^* = -.07  F(4, 27) = 0.48  *p* = .749 | | | | | | |  | | Adj. *R^2^* = -.07  F(4, 25) = 0.56  *p* = .695 | | | | | |  | | Adj. *R^2^* = -.10  F(4, 27) = 0.31  *p* = .871 | | | | | |
|  |  |  |  |  |  |  |  |  |  |  |  |  |  |  |  |  |  |  |  |  |  |  |  |  |  | |  |  |  |  |  |  |  | |  |  |  |  |  |  |
|  |  |  |  |  |  |  |  |  |  |  |  |  |  |  |  |  |  |  |  |  |  |  |  |  |  | |  |  |  |  |  |  |  | |  |  |  |  |  |  |
|  |  | **Expected nocebo effects** | | | | | | | | |  |  | |  | |  | | |  |  | |  | |  | |  | |  | |  | |  | |  | |  | |  | |  |
|  |  | **Induction of cowhage itch** | | |  | **Generalization to mechanical sensations** | | |  |  | | | | |  | |  |  | | | | |  | |  | |  | |  | |  | |  | |  | |  | |  | |
|  |  | *β* | *SE_a_* | 95%CI |  | *β* | *SE_a_* | 95%CI |  |  | | |  | |  | |  |  | | |  | |  | |  | |  | |  | |  | |  | |  | |  | |  | |
| **Experiment 2** |  |  |  |  |  |  |  |  |  |  | | |  | |  | |  |  | | |  | |  | |  | |  | |  | |  | |  | |  | |  | |  | |
|  | **Anxiety** | .01 | .10 | -.16, .23 |  | .06 | .08 | -.14, .17 |  |  | | |  | |  | |  |  | | |  | |  | |  | |  | |  | |  | |  | |  | |  | |  | |
|  | **Stress** | .04 | .07 | -.10, .16 |  | -.03 | .06 | -.12, .10 |  |  | | |  | |  | |  |  | | |  | |  | |  | |  | |  | |  | |  | |  | |  | |  | |
|  | **Itch catastrophizing** | .01 | .05 | -.10, .09 |  | .05 | .04 | -.03, .14 |  |  | | |  | |  | |  |  | | |  | |  | |  | |  | |  | |  | |  | |  | |  | |  | |
|  | **Attention to itch** | -.00 | .03 | -.06, .06 |  | -**.06** | .03* | -.12, -.02 | | | | |  | |  | |  |  | | |  | |  | |  | |  | |  | |  | |  | |  | |  | |  | |
| Full model | | Adj. *R^2^* = -.09  F(4, 39) = 0.14  *p* = .966 | | |  | Adj. *R^2^* = .06  F(4 39) = 1.72  *p* = .166 | | |  |  | | |  | |  | |  |  | | |  | |  | |  | |  | |  | |  | |  | |  | |  | |  | |
|  |  |  |  |  |  |  |  |  |  |  | | |  | |  | |  |  | | |  | |  | |  | |  | |  | |  | |  | |  | |  | |  | |
|  |  |  |  |  |  |  |  |  |  |  | | |  | |  | |  |  | | |  | |  | |  | |  | |  | |  | |  | |  | |  | |  | |

*Note:*p<0.05. β* is the standardized regression coefficient. n/a, not applicable. *SEa*, bootstrapped standard error of mean. CI, bootstrapped confidence interval.
